# Attenuation of tacrolimus induced oxidative stress, mitochondrial damage, and cell cycle arrest by *Boerhavia diffusa* root fraction in mdck cell lines 

**DOI:** 10.22038/ijbms.2021.56519.12618

**Published:** 2021-08

**Authors:** Kalaivani M.K, Cordelia Mano John, Bhavana Jonnagaladda, Akila Kesavan, Sumathy Arockiasamy

**Affiliations:** 1 Department of Biomedical Sciences, Sri Ramachandra Institute of Higher Education and Research (DU), Porur, Chennai 6000116, India; 2 Department of Human Genetics, Sri Ramachandra Institute of Higher Education and Research (DU), Porur, Chennai, 600116, India

**Keywords:** Apoptosis, Boerhavia diffusa, Caspase 3, Cell cycle, HPLC, Nephrotoxicity, Quercetin, Reactive oxygen species

## Abstract

**Objective(s):**

The protective effect of ethyl acetate fraction (EAF) of *Boerhavia diffusa* roots against Tacrolimus (TAC) induced nephrotoxicity was studied using MDCK cell lines.

**Materials and Methods:**

Ethanolic root extract of *B. diffusa *was fractionated using the liquid-liquid partition method. The cytotoxic effect of TAC and protective effect of EAF co-treatment were studied in MDCK cell lines by measuring ROS, LPO, and NO levels; collagen accumulation, effect on mitochondrial membrane integrity and cell cycle analysis were studied. The active component in EAF was quantified by HPLC analysis.

**Results:**

TAC induced toxicity, leading to apoptosis and necrosis, was significantly reduced (*P*<0.001) in EAF co-treatment, with reversal of cell cycle arrest and reduced cell population at sub G0/G1 phase. Further, ROS (*P*<0.05), LPO and NO (*P*<0.001), were significantly reduced with EAF co-treatment compared with TAC individually treated cells. TAC induced mitochondrial membrane integrity loss was found to be significantly reduced in co-treated cells, as measured by rhodamine123 (*P*<0.05) and translocation of cytochrome c (*P*<0.001) from nucleus to cytoplasm, and caspase 3 release (*P*<0.001). The same was confirmed through annexin-FITC and PI staining (*P*<0.05) with reduced apoptotic and necrotic death in co-treated population. Interestingly, EAF co-treatment decreased collagen accumulation (*P*<0.001) with significant increase in the cell survival of tubular epithelial cells. HPLC analysis showed the presence of Quercetin (87.5 mg/g) in EAF, which may be responsible for the nephroprotective role.

**Conclusion:**

Thus, these results provide sound evidence that EAF may be an effective adjuvant therapy to prevent nephrotoxicity induced by TAC.

## Introduction

The calcineurin inhibitor (CNI), cyclosporine, in human kidney transplantation was introduced in late 1970s which revolutionized the transplantation medicine and made transplantation a preferable therapeutic intervention for end-stage renal diseases (1). In 1984, another CNI, Tacrolimus (FK506), a peptide macrolide antibiotic isolated from the fungus *Streptomyces tsukubaensis*, was discovered, which was successfully used in liver, kidney, and heart allograft transplantation. The mode of action of these CNIs involves inhibition of calcineurin, a calcium- and calmodulin-dependent phosphatase. The binding of these drugs to calcineurin inhibits its phosphatase activity, which suppresses the transcription of IL-2 and T cell activation, via inhibition of dephosphorylation and impaired translocation of the nuclear factor of activated T cells (NFAT) (1, 2). Tacrolimus (TAC) gained popularity due to improved graft survival at a low dose and has become an integral part of immunosuppressive regimen over the years (26% in 1998 to 92.3% in 2014) (3). Despite its increased efficacy as an immune-suppressor, the calcineurin inhibition pathway was also found to rise toxicity beyond immunosuppression, as CNIs are not T-cell specific, including nephrotoxicity, cardiac damage, high blood pressure, which limits its clinical application (1, 4). 

The mechanism of TAC-induced nephrotoxicity is obscure, but literature review suggests that generation of reactive oxygen species (ROS), leading to increased oxidative stress, inflammation as well as disruption of antioxidant defense genes such as superoxide dismutase (SOD), catalase (CAT), glutathione reductase (GR) (5), results in cell injury and death (6). TAC was shown to induce apoptosis in tubular cells by activation of the mitochondrial pathways and caspases (7). It is also shown to up-regulate the expression of intra-renal transforming Growth Factor β (TGF-β) aggravating interstitial fibrosis (8). Hence, long-term TAC treatment is coupled with severe nephrotoxicity leading to end stage renal disorder (ESRD). Therefore, it has become inevitable to identify new pharmacological therapy, using knowledge of traditional medicines, to ameliorate nephrotoxicity without compromising the clinical usage of TAC in long-term survival of allografts. Many natural products have already been investigated for their protective role against TAC-induced nephrotoxicity, which is mainly attributed to their antioxidant property to reduce the oxidative stress (9-12).

*B. diffusa, *a perennial creeper commonly known as *Punarnava*, belonging to the *Nyctaginaceae* family, is widely used in Indian system of medicine. Pharmacological studies have already shown diuretic, anti-nephrotic, anti-inflammatory, anti-nociceptive, anti-convulsant, immunomodulatory and antioxidant properties of the plant (13). The root of this plant was studied for its protective role against various chemotherapeutic drugs, such as cisplatin, gentamicin, and mercuric chloride induced nephrotoxicity (14-20). A previous study in our laboratory has shown the protective role of ethyl acetate fraction (EAF) of the root of the plant against Cyclosporine A, a CNI, induced nephrotoxicity, attributed to the comparative reduction in ROS generation and inhibition of apoptosis (21). Therefore, the present study is aimed to understand the activity of EAF against another CNI, TAC-induced nephrotoxicity in MDCK cells.

## Materials and Methods

***Chemicals and reagents***

Propidium iodide (PI), bisBenzimide (Hoechst staining), acridine orange/ethidium bromide, and rhodamine123 were purchased from Sigma Aldrich, India. Caspase 3 and Cytochrome c were purchased from Santa Cruz Biotech. TAC was purchased from Selleck Chemicals Pvt limited, USA. Antibiotics & antimycotics, fetal bovine serum (FBS), Dulbecco’s Modified Eagle’s Medium (DMEM), and other routine chemicals were purchased from Himedia, India.

***Cell culture***

The epithelial origin distal tubular cell line, Madin-Darby Canine Kidney (MDCK) was procured from National Centre for Cell Sciences, Pune, India. The cells were maintained in DMEM containing 4.5 g/L D-glucose, supplemented with 10% FBS in a humidified 5% CO2 incubator at 37 °C. All studies were performed in the MDCK cell lines.

***Extraction & fractionation of B. diffusa***

The extraction and fractionation of the EAF of the plant were performed as mentioned in Kalaivani *et al*. 2018 (21). 

***Cell viability assays***

*MTT assay*

The effects of Crude Extract (CE) and its fractions against TAC-induced cell toxicity were determined by MTT (3-(4,5-dimethylthiazol-2-yl)-2,5-diphenyltetrazolium bromide) assay (22). The cells were treated with TAC, alone as well as in combination with CE & various fractions (10, 25, 50, 75, and 100 µg/ml). Following 24 hr exposure, 20 µl of MTT (5 mg/ml) was added and incubated at 37 °C with 5% CO_2 _for 2 hr. The formazan crystals formed were dissolved in DMSO and the absorbance was measured at 570 nm in a spectrophotometer. 

*Neutral red (NR) uptake assay*

The lysosomal viability of the treated cells was studied with the cationic dye NR reagent. The treated cells were washed with pre-warmed PBS and 125 µl of NR was added, at a final concentration of 50 µg/ml and incubated for 3 hr. Following incubation, the wells were washed with PBS, and 100 μl of NR destaining solution (1% (v/v) acetic acid, 50% (v/v) ethanol, and 49% (v/v) distilled water) was added. The absorbance was read at 540 nm to quantify the amount of retained dye in treated cells (23).

*Lactate dehydrogenase (LDH) release*

A common technique for determining cytotoxicity involves measuring the activity of the cytoplasmic enzyme, lactate dehydrogenase (LDH), released by the damaged cells. LDH is rapidly released into the cell culture medium when the cell membrane is damaged due to apoptosis or necrosis and the amount of LDH released signifies the magnitude of cell membrane damage (24). The free LDH released into the culture media and intracellular LDH from cellular lysate of the treated cells were prepared by treating with 1% Triton X-100 for 10 min. The cells were treated with 1% Triton X-100 solution for 5 min for maximum LDH release and were used as a control. The clear supernatant obtained from centrifuging at 250xg, 4 °C for 10 min, was used for quantification of LDH release. The percentage of LDH release represents the cytotoxicity of a compound. 

*Clonogenic assay*

The treated cells were trypsinized and approximately 100 cells were cultured in complete medium and observed for colony formation. After two weeks, the cell colonies were fixed in 10% neutral buffered formalin solution and stained with crystal violet dye. The accumulated intracellular dye was eluted with 50% sodium citrate and the absorbance was measured at 595 nm using a spectrophotometer (25).

*Morphological staining of apoptotic & necrotic cells*

The apoptotic cell morphology of the treated cells was observed by Hoechst stain and AO/EtBr staining while the necrotic morphology was evaluated using propidium iodide (PI). Briefly, the treated cells were fixed in 70% ethanol for 10 min and air dried, and then stained with 2 µM Hoechst (26). Double staining was performed for AO/EtBr staining (1 part of 100 µg/ml AO and 1 part of 100 µg/ml EtBr in PBS) to 25 µM cell suspension and examined under the fluorescent microscope and photographed (27). For PI staining, the cells were stained with 10 µM PI and observed under a Nikon Eclipse inverted fluorescent microscope (100x) (28).

***DNA fragmentation***

Intra-nucleus DNA fragmentation was assessed by isolating the genomic DNA from both attached and floating cells (29). Treated cells were disrupted with 1% SDS and incubated overnight with proteinase K at 48 °C. The DNA was isolated with phenol: chloroform and precipitated with ethanol. The DNA pellet was dissolved in Tris-EDTA (TE) buffer and incubated with RNase A for 1 hr at 37 °C. The resolved DNA was stained in Tris-borate/EDTA buffer with ethidium bromide to observe the fragmented DNA.

***Cell cycle distribution by FACS analysis***

The treated cells were washed with ice-cold phosphate buffer saline (PBS) and disrupted in hypotonic solution (1% sodium citrate, 0.04% RNAse, 0.05% PI, 3%Triton X-100). The percentage of cells in different phases of cell cycle was determined by C6 Accuri flow cytometer (30).

***Quantification of apoptotic and necrotic cells***

To analyze the percentage of apoptotic and necrotic cells, Annexin V and PI staining was performed using the commercial assay kit (Invitrogen). The apoptotic and necrotic cells in the treated population were quantified according to the manufacturers’ stipulated protocol. 

***Measurement of mitochondrial membrane potential (*** ***ΔΨm*** ***)***

Depolarized mitochondrial membrane potential was assessed by Rhodamine 123 staining, a cationic dye that accumulates in both the inner membrane and matrix space (31). The membrane damage affects the dye uptake, leading to less fluorescence in the cells. The treated cells were incubated with the dye for 30 min and washed with ice-cold PBS to remove the excess dye. The cells were scraped and centrifuged at 2000 rpm for 10 min. The pellet was resuspended in 100 µl of PBS and the cells were analyzed by a BD C6 Accuri flowcytometer (30). 

***Caspase release assay***

Caspase 3 activity was measured using EnzChek Caspase Assay Kit (Molecular Probes). The treated cells were quantified for the Caspase 3 release as per the manufacturer’s protocol.

***Western blotting***

The treated cells were lysed in Radioimmunoprecipitation assay (RIPA) buffer and protein concentration was estimated by Lowry’s method. The cell lysate was electrophoresed in 12% SDS polyacrylamide gel and transferred into PVDF membranes. The membranes were incubated with primary antibodies of cytochrome c (1:2000) in Tris-buffered saline and then washed and further incubated with Horseradish peroxidase (HRP) conjugated anti-mouse IgG (1:5000) and goat-anti-rabbit IgG (1:5000). Protein bands were detected using a chemiluminescence system (ECLKit) and quantified in Chemi Doc XRS Imaging System, Bio-Rad (USA).

***ROS measurement***

ROS production was measured with the cell-permeable fluorogenic dye, 2,7-dichlorodihydrofluorescein diacetate (DCF-DA), using flowcytometry (2). Treated cells were trypsinized and preloaded for 20 min with 20 μM freshly prepared DCF-DA in ethanol. The cells were analyzed by flowcytometry with excitation at 475 nm and emission at 525 nm in a BD C6 Accuri flowcytometer (28).

***Lipid peroxidation (LPO) measurement***

LPO is a non-enzymatic process of the oxidative deterioration of lipids, in which ROS attack lipids in an uncontrolled manner leading to elevated membrane rigidity and structural impairment of the membranes (32). LPO was measured in terms of malondialdehyde (MDA). Following treatment, the cells were scraped and washed twice in PBS and lysed with 1.15% potassium chloride for 30 min. The contents were centrifuged at 5000 rpm and 500 µl of the supernatant was incubated with 2 ml of 0.1% TBA for 15 min at 100 °C. After cooling, the mixture was centrifuged at 1000 rpm for 10 min and the supernatant was again incubated with 1 ml of 0.8% TBA for 15 min at 100 °C. Absorbance was measured at 532 nm using a spectrophotometer. The concentration of MDA-TBA complex in the treated cells was calculated from the standard curve of MDA (2-10 µM) (33). 

***Nitric oxide (NO) release***

NO was measured in terms of nitrite formed, using Griess reagent (34)**. **NO released into the cell culture medium in the treated as well as the control cells was measured by adding the reagent to the cell supernatants. The absorbance was measured at 548 nm and compared with the standard NaNO_2 _(20- 100 µM).

***Collagen staining***

Following treatment, the cells were washed with PBS, fixed with Bouin’s fluid and stained with Direct Red O. The stained cells were photo-documented and the accumulated dye was extracted using 0.1 N NaOH and the absorbance was measured at 550 nm (35).

***Quantification of quercetin in EAF using HPLC***

HPLC analysis was performed in C18 column (5 µm, 20 mm x 4.6 mm). The mobile phase was a mixture of methanol: H_2_O: ortho-phosphoric acid (100:100:1), employed at a flow rate of 1 ml/min. The column temperature was maintained at 30 °C, and the standard flow rate was maintained at 1 ml/min. The stock solution was prepared by dissolving 10 mg of standard and sample in 100 ml of HPLC grade methanol. The sample solution was filtered through a 0.45 µm PVDF filter membrane and the mobile phase was degassed before injecting. 10 µl sample was injected and the eluted sample was detected at 370 nm. 

***Statistical analysis***

Results were expressed as mean±S.D. of three independent experiments. Statistical analysis was performed using one-way ANOVA and Tukey’s *post-hoc* multiple comparison tests using the Graph Pad Prism (7.0) program (Graph Pad Software Inc., San Diego, CA, USA). A *P*-value of <0.05 between the TAC treated group and other groups was considered statistically significant (*** denotes *P*<0.0001; ** denotes* P*<0.001; * denotes* P*<0.05).

## Results

***Effect of B. diffusa extracts and its fractions on TAC induced cytotoxicity***

**Figure 1:**
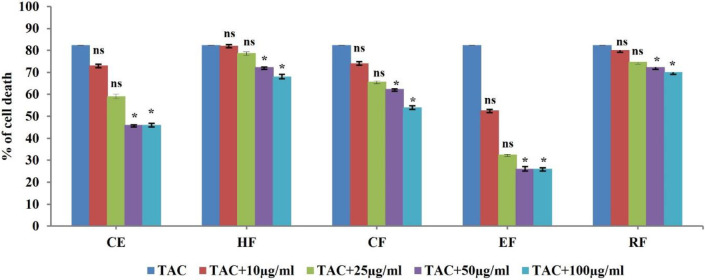
Effect of *Boerhavia diffusa* crude extract and its fractions on TAC induced nephrotoxicity. The effect of TAC and co-treatment with crude extract and other fractions was estimated by MTT assay. CE: Crude Extract, HF: Heaxane Fraction, CF: Chloroform Fraction, EF: Ethyl Acetate Fraction, RF: Residual fraction. Statistical comparison is between TAC (control) vs treated. ns – not significant, *P<0.05

TAC induced cytotoxicity was individually studied in MDCK cell lines, and among the various concentrations (data not shown), 50 µM TAC was shown to induce 82.41% cell death following a 24-hr exposure (*P*<0.0001). Hence further studies were performed with the same concentration. The effect of TAC (50 µM) individually and co-treatment with *B. diffusa *crude extract (CE) and hexane, chloroform, ethyl acetate, and residual fractions on the viability of MDCK cells was evaluated by MTT assay. EAF, compared with the other fractions, was found to reduce TAC induced cytotoxicity in the MDCK cell lines, with more than 70% cell viability obtained at 25, 50, and 100 µg/ml (Figure 1). Therefore, further studies were performed with EAF (25 and 50 µg/ml). 

***Protective effect of EAF against TAC induced cytotoxicity***

**Figure 2:**
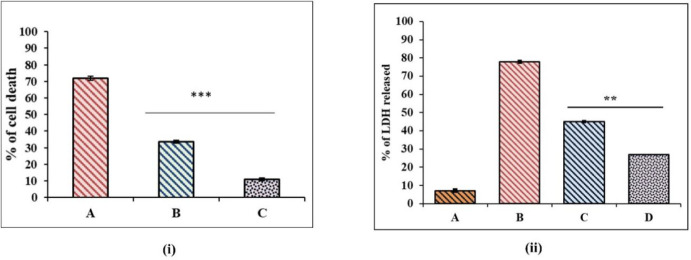
Cytoprotective effect of ethyl acetate fraction (EAF) against tacrolimus (TAC) induced toxicity. (i) Effect of EAF against TAC-induced toxicity studied by neutral red uptake assay. EAF (25 & 50 μg/ml) co-treatment reduced TAC induced cell death in MDCK cell lines. (A) TAC (50 µM), (B) TAC (50 µM) + EAF (25 μg/ml), (C) TAC (50 µM) + EAF (50 μg/ml). (ii) Comparative evaluation of LDH release in TAC and EAF co-treated cells. Extent of LDH release was reduced in EAF treated groups adose dependently, compared with TAC individually treated cells (*P*<0.001). (A) Control, (B) TAC (50 µM), (C) TAC (50 µM) + EAF (25 μg/ml), (D) TAC (50 µM) + EAF (50 μg/ml). Data are expressed as mean±SEM (n= 3). (***P<0.0001, **P<0.001 vs control)

The protective effect of EAF on TAC induced nephrotoxicity was also analyzed by neutral red uptake and LDH release assay. The cells were co-treated with TAC (50 µM) and EAF (25 & 50 µg/ml) and incubated for 24 hr. The neutral red assay showed increased cell viability in EAF co-treated cells compared with TAC alone (*P*<0.0001) (Figure 2 (i)). An assessment of LDH enzyme leakage from the cells, which demonstrates the cytotoxic effect, was also performed for TAC and EAF co-treated cells. The LDH release in TAC treated cells was found to be 78.04%±0.085, while it has been significantly (*P*<0.001) reduced to 45.06%±0.26 (25 µg/ml) and 27.03%±0.32 (50 µg/ml) in EAF co-treated cells (Figure 2(ii)). 

***Effect of EAF on cell survival rate of TAC treated cells***

**Figure 3:**
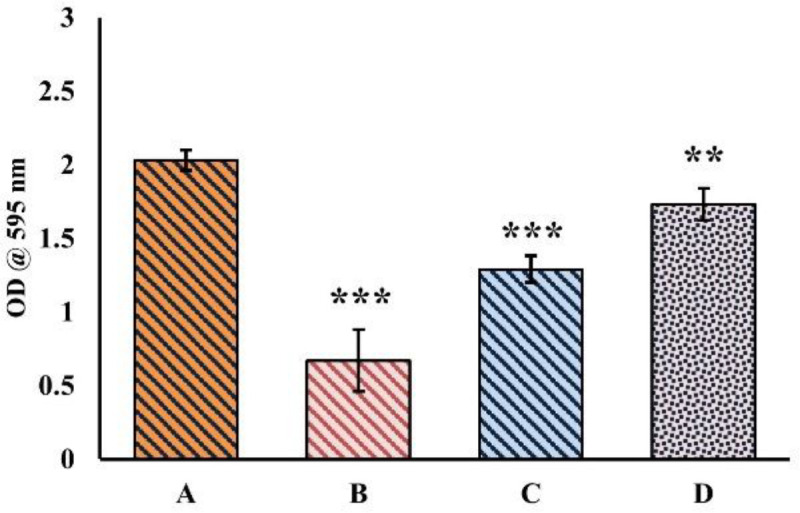
Effect of EAF on cell survival rate of tacrolimus (TAC) co-treated cells. Ethyl acetate fraction (EAF) co-treatment significantly increased growth potential (*P*<0.001) in terms of colony formation. (A), Control (B) TAC (50 µM), (C) TAC (50 µM) + EAF (25 μg/ml), (D) TAC (50 µM) + EAF (50 μg/ml)

Cell survival rate was studied through the ability of cells to form colonies after treatment with TAC alone and with EAF (25 & 50 μg/ml). TAC treated cells exhibited low survival rate with reduction in clonogenic potential. However, this was found to be improved distinctly (*P*<0.001) with EAF co-treatment, where the cells were able to form colonies well, which indicates the better survival rate of the cells (Figure 3).

***Apoptosis and necrosis induced morphological changes***

**Figure 4:**
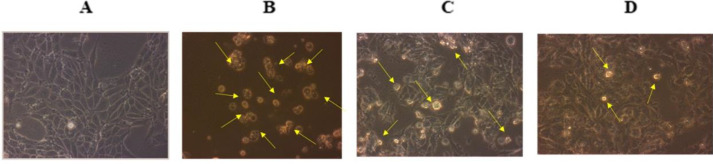
Morphological changes in the TAC & EAF co-treated cells observed under Phase contrast microscope. Cells were photographed at 100x. TAC treatment showed increased cell shrinkage and reduced cell density (yellow arrow), while EAF co-treatment decreased cell shrinkage and increased cell density. (A) Control, (B) tacrolimus (TAC) (50 µM) treated, (C) TAC (50 µM) + EAF (25 μg/ml), (D) TAC (50 µM) + Ethyl acetate fraction (EAF) (50 μg/ml)

**Figure 5:**
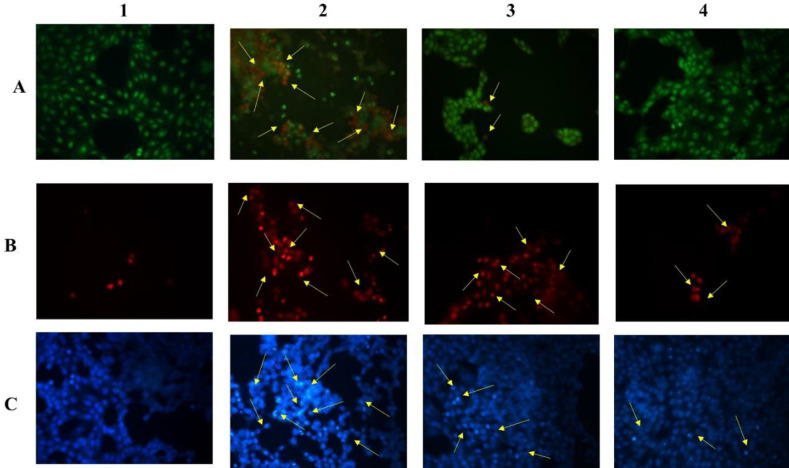
Apoptotic and necrotic changes in tacrolimus (TAC) and Ethyl acetate fraction (EAF) Co-treated cells. The cells were stained with AO/EtBr, PI & Hoechst and photographed in a fluorescent microscope (100x). (A) AO/EtBr staining revealed late apoptosis (A2-orange) and necrosis (A2-red) in TAC (50 µM) alone treated cells and an increase in live cell population in EAF (A3&4) co-treated cells. (B) PI staining showed more necrosis (red) in TAC-treated (B2) cells, with a comparative reduction in EAF (B3 & 4) co-treated cells. (C) Hoechst staining showed apoptotic morphology in TAC (C2) treated cells, which was diminished in EAF (C3 & 4) co-treated groups. (1) Control, (2) TAC (50 µM), (3) TAC (50 µM) + EAF (25 μg/ml), (4) TAC (50 µM) + EAF (50 μg/ml)

**Figure 6:**
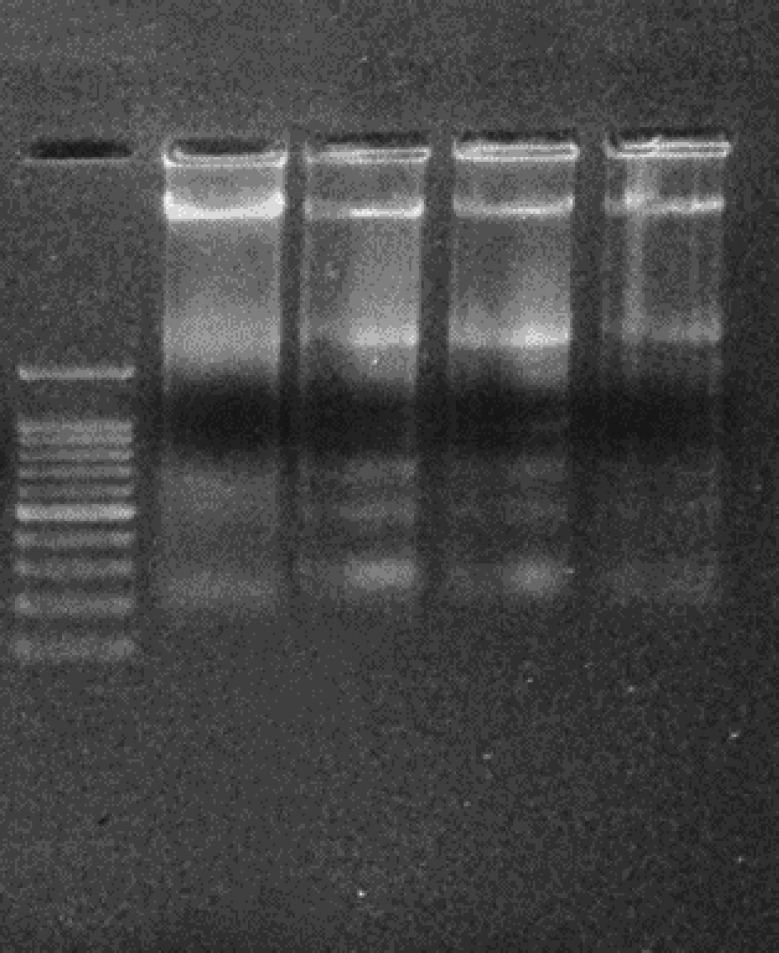
DNA Fragmentation analysis in tacrolimus (TAC) and Ethyl acetate fraction (EAF) co-treated cells. Electrophoresis of the isolated DNA from TAC alone and EAF co-treated cells showed intranucleosomal DNA degradation in TAC-treated cells, but the intensity of the bands was relatively reduced in EAF co-treated groups. (M) Marker (a) control, (b) TAC (50 µM), (c) TAC (50 µM) and EAF (25 μg/ml), (d) TAC (50 µM) and EAF (50 μg/ml)

The morphological changes in the control as well as TAC treated cells with and without EAF were observed and photographed under a Phase contrast microscope (Nikon Eclipse T*i*-S). TAC treated cells showed cell shrinkage and reduced cell density, whereas the EAF co-treated cells showed increased cell viability with a reduction in cell shrinkage (Figure 4). Furthermore, the apoptotic and necrotic changes in TAC and EAF co-treated cells were also studied with acridine orange/ethidium bromide (AO/EtBr), PI and Hoechst stain and observed under fluorescent microscopy (Nikon Eclipse T*i*-S, Japan) and photographed (10x) using Image-Pro® Plus software. AO/EtBr staining presented both early and late apoptotic as well as necrotic morphology in TAC alone treated cells, while EAF co-treatment considerably minimized the apoptosis and necrosis cell population (Figure 5A). Similarly, PI-positive necrotic cells were found to be more in TAC treated cells, which were notably reduced in EAF co-treated cells (Figure 5B). Hoechst staining was also performed to study the apoptosis, which is principally characterized by cell rounding, chromatin condensation, and nuclear fragmentation. These changes were found to be higher in TAC treated cells and comparatively declined in EAF co-treated cells (Figure 5C). Furthermore, intra-nucleosomal DNA degradation, an integral part of apoptosis, which was observed in TAC alone treated cells, was also significantly reduced in EAF co-treated cells, dose-dependently (Figure 6).

***Cell cycle analysis by Flow cytometry***

The nephroprotective effect of EAF against TAC-induced cytotoxicity was further evaluated by investigating the cell cycle profiles of the treated cells by flowcytometry. The analysis of DNA content at various cellular phases revealed that TAC treatment induced a three-fold increase in cell accumulation at S-phase compared with control. Additionally, TAC treatment also showed a fivefold increase in cell population at sub G_0_/G_1_ phase, compared with control, which is indicative of drug toxicity. Conversely, EAF co-treatment showed a significant reduction in cell population at sub G_0_/G_1_ phase, as well as preventing the cell cycle arrest at S phase, thus accumulation, which is an indicative of reduced cytotoxicity (Table 1).

***Quantification of apoptotic and necrotic in TAC & EAF Co-treated cells***

**Figure 7:**
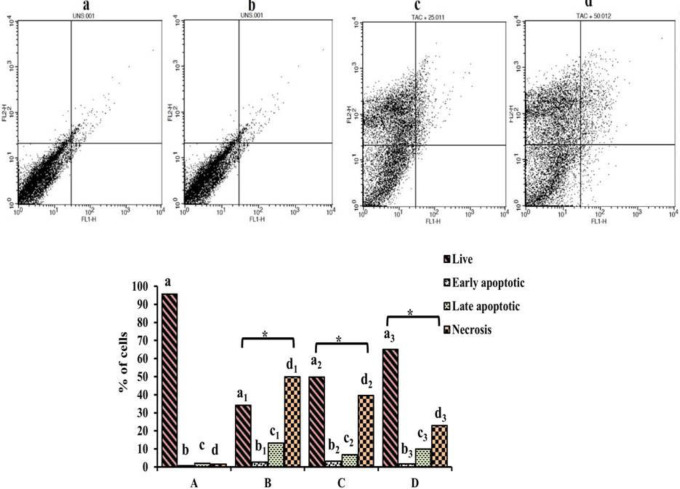
Flowcytometric analysis of apoptotic and necrotic cell population. (i) Flow cytometric analysis of distribution of cells stained with Annexin V-FITC/PI. (ii) Percentage of live, early, and late apoptotic and necrotic cells stained with Annexin V-FITC/PI staining. (A/a) Control; (B/b) tacrolimus (TAC) (50 µM) alone treated; (C/c) TAC (50 µM) + EAF (25 μg/ml); (D/d) TAC (50 µM) + Ethyl acetate fraction (EAF) (50 μg/ml). Data are represented as mean ± SEM (n = 3). Statistical comparison is between a (control) vs a1, a2, and a3 (treated) and so forth. Difference in subscripts denote statistical significance of *P*<0.05

Cellular apoptosis and necrosis induced by TAC were quantitatively measured by Annexin V-FITC/PI staining. In the apoptosis map, TAC treatment showed 50% necrotic cells (stained with PI), 13.20% late apoptotic (stained with both Annexin V-FITC and PI), 2.65% early apoptotic cells (stained only with Annexin V-FITC) and 34% live cells (unstained), while EAF co-treatment decreased the necrotic cells (23%) and increased the live cells (65%) (*P*<0.05) significantly (Figure 7), which indicates the protective role of EAF against TAC induced apoptotic and necrotic cell death. 

***Attenuation of intrinsic apoptotic pathway & mitochondrial damage in EAF co-treated cells***

**Figure 8:**
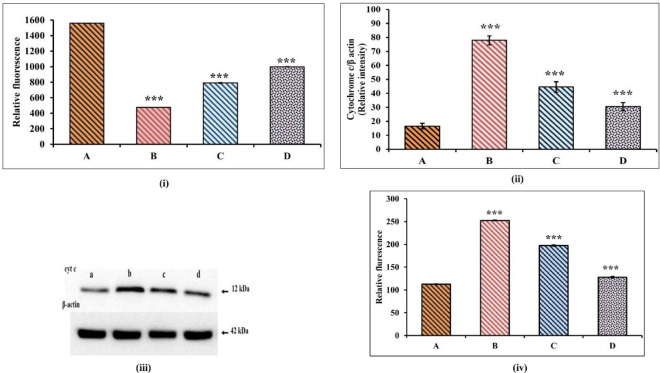
Attenuation of tacrolimus (TAC) induced intrinsic apoptosis and mitochondrial damage by Ethyl acetate fraction (EAF)

The loss of mitochondrial membrane integrity in TAC and EAF co-treated MDCK cells was assessed by selective uptake of rhodamine dye (*P*<0.05) (Figure 8(i)). Mitochondrial damage concomitantly results in the translocation of cytochrome c from nucleus to cytosol (*P*<0.001), activating caspase 3 release (*P*<0.001) as observed in TAC & EAF co-treated cells (Figures 8(ii) & 8(iii)). This indicates the detrimental effect of TAC mitochondrial membrane integrity on MDCK cells, leading to induction of severe apoptosis. However, an increased rhodamine uptake was observed in EAF co-treated cells, which authenticate the protective role of EAF in stabilizing the mitochondrial membrane potential and preventing the translocation of cytochrome c to cytosol, thereby consequently inhibiting caspase 3 release and apoptosis (Figure 8 (iv)).

***Effect of EAF on TAC induced oxidative stress***

**Figure 9:**
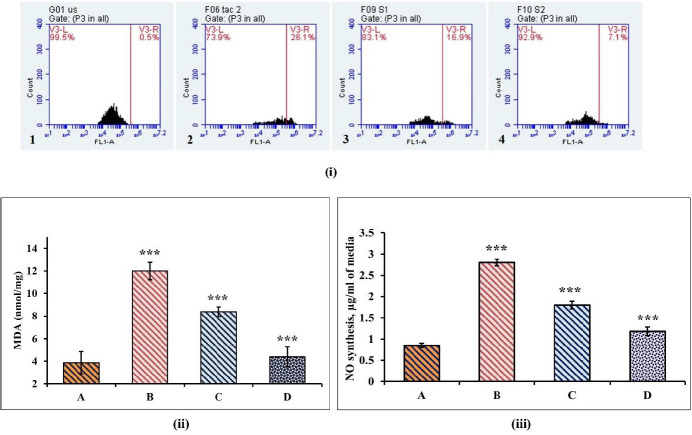
Protective effect of Ethyl acetate fraction (EAF) against tacrolimus (TAC) induced oxidative stress

TAC induced oxidative stress in MDCK cells was measured by flowcytometry using DCFH-DA staining. The results showed an increase in number of DCF positive cells (26.09% ± 0.06) in the TAC alone treated groups, which was found to be comparatively decreased in EAF co-treated groups (*P*<0.05) (Figure 9 (i)). Moreover, exposure to TAC resulted in an increase in the MDA and NO content in the cells, compared with the control. But EAF co-treatment significantly reduced MDA and NO content in a concentration-dependent manner (*P*<0.0001) (Figures 9 (ii) & 8 (iii)). 

***EAF reduces collagen accumulation and increases cell survivability***

**Figure 10:**
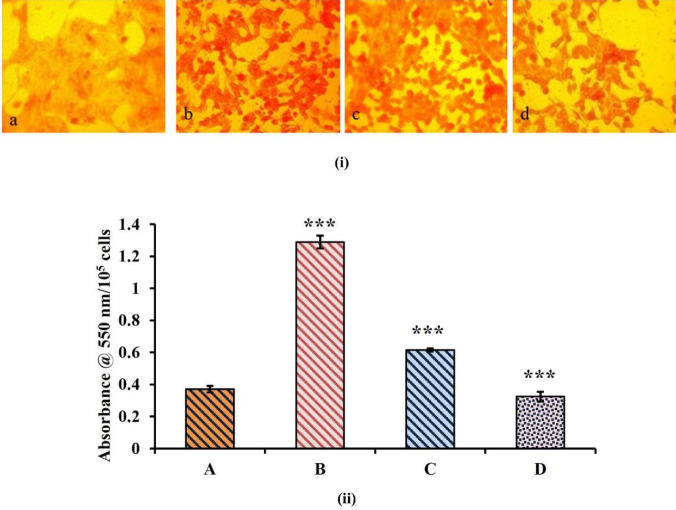
Collagen staining and quantification using direct red O stain

Collagen is the major constituent of extra-cellular matrix protein, and the intra-renal expression of this protein was found to be highly elevated in TAC induced nephrotoxicity. However, EAF co-treatment resulted in significant reduction in collagen accumulation (*P*<0.0001). This reduction in collagen accumulation was quantitatively measured using the direct red-O stain. The intensity of dye accumulated was found to be proportionately less in the EAF co-treated groups compared with TAC treated cells (Figure 10). 

***HPLC analysis of quercetin***

**Figure 11:**
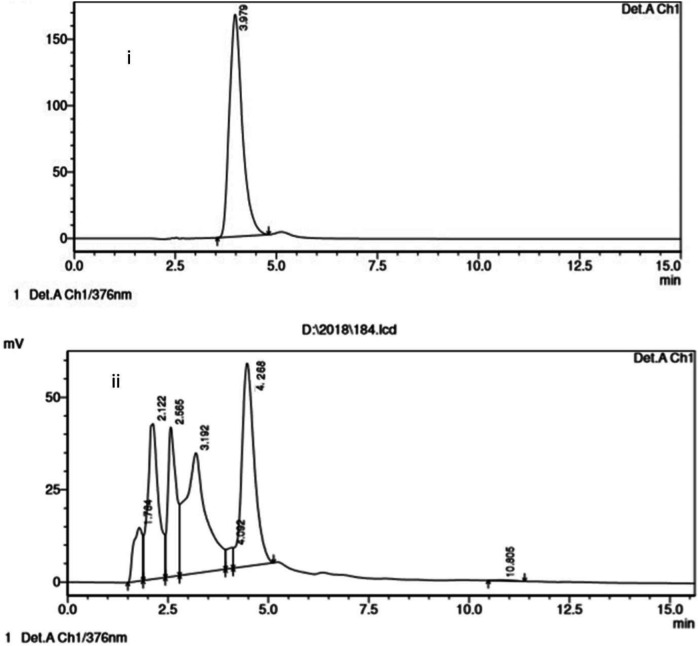
Chromatogram of HPLC

HPLC analysis was performed to quantify major phytoconstituents in EAF responsible for its protective role against TAC induced toxicity. The major constituent was found to be Quercetin, with 87.5 mg/g in the fraction (Figure 11).

## Discussion

CNI, the immunosuppressants, inhibit the calcineurin, a calcium-calmodulin-dependent phosphatase, leading to inactivation of T-cell activation (11). Calcineurin is expressed at high levels in various organs including the kidney and performs critical processes such as T cell activation, angiogenesis, cardiac hypertrophy, and muscle and neural development. Thus, inhibition of calcineurin is not specific to the immune cells and may cause harmful effects in a diverse array of pathological conditions (36). The metabolism of CNI mainly occurs in the liver, gut and kidney (37), where the kidney is one of the main target organs for drug toxicity due to its high perfusion rate and specialized uptake system (38). The pathological factor associated with TAC-induced nephrotoxicity is attributed to excessive production of ROS and oxidative stress, resulting in apoptosis in kidney tubular cells, which has been verified in previous studies (5, 37, 39).

The plant extracts and phytocompounds with various active ingredients and multiple actions have shown beneficial activity against drug-induced nephrotoxicity (38). Previous studies in our laboratory with *B. diffusa *root extract, have shown a protective role, against the CNI, cyclosporine A (CsA) and cisplatin (CP) induced toxicity in MDCK cells, by significantly reducing the ROS induced MDCK cell apoptosis (19, 21). Therefore, the present study was undertaken to evaluate the protective role of EAF of *B. diffusa* root against CNI, TAC induced nephrotoxicity in MDCK cell lines. 

In the present study, among other fractions, EAF was found to increase the survivability of MDCK cells, dose-dependently, in the presence of TAC. A previous study with CsA-induced nephrotoxicity has shown increased ROS generation in the mitochondria, leading to cytochrome c release into the cytosol, activation of caspase, and apoptosis (33). The current study also evidenced similar results, where TAC treatment increased ROS levels, damaging the mitochondrial membrane potential, leading to activation of caspase 3 and apoptosis in MDCK cell lines. 

A small rise in apoptotic rate in the kidney tubular cells causes sizeable loss of cells, as the half-life of tubular cells is around 1–2 hr. However, the cell death signalling pathway can be counteracted by cell survival signals, which is mainly dependent on the balance between caspase activation by the target drug and the ability of the cells to block the caspase pathway (7), by modulating the cell cycle regulators, a promising approach to treat nephrotoxicity (25). Thus, in the present study, EAF co-treatment effectively reduced TAC induced DNA damage and attenuated the intrinsic apoptotic pathway by decreasing the mitochondrial membrane damage and preventing the translocation of cytochrome c, thereby increasing the cell survival. Furthermore, G1/S checkpoint plays a crucial role in the regulation of cell proliferation, and cell cycle arrest at this point results in an abnormal growth or apoptosis (40). Thus, TAC induced S phase cell cycle arrest in MDCK cells, activating apoptosis, was counteracted by EAF co-treatment, preventing the cell cycle arrest concentration-dependently. These results are in correlation with the previous study, where ethanolic extract of whole plant of *B. diffusa* reduced ROS production in the mitochondria and maintained the mitochondrial membrane potential (41). 

Mitochondria play an essential role in the cytotoxicity of several drugs and chemicals (11, 42). In the present study, ROS-dependent mitochondrial dysfunction was shown to play a pivotal role in the induction of apoptosis in MDCK cells. Increased ROS levels attack the membrane phospholipids, causing mitochondrial membrane depolarization, which also impairs the cellular redox status, leading to mitochondrial dysfunction and activation of caspase cascade resulting in apoptosis (37, 38). Studies with CNI have shown increased ROS induced oxidative stress, where TAC induced ROS generation was shown to damage the cellular components, including lipids, proteins, and DNA (38, 43).

TAC elevates NO production, which reacts with superoxides to form a highly reactive and a dangerous peroxidant, peroxynitrite (ONOO-), involved in renal cell damage (44). LPO, produced by free radicals, is considered an excellent oxidative stress biomarker, which damages the cell membrane (42). The results of the current study are in correlation with these findings, where increased levels of ROS, LPO, and NO were observed in the TAC treated cells. MDA, a secondary product of LPO, elevated in TAC treated cells, was found to be responsible for morphological and functional deterioration of renal cells (45). The study has shown a significant rise in LPO levels dose-dependently in TAC treated MDCK cells, however, co-treatment with EAF has shown a reduction in the LPO levels, retaining cell integrity. Since an effective redox balance maintains the mitochondrial function, *B. diffusa* with excellent anti-oxidant properties may regulate the redox status, a potential in renal cell function. These results agree well with those of previous studies, which showed that inhibition of TAC-induced apoptosis was associated with the suppression of ROS production (7, 46, 47) 

Prolonged intake of TAC was found to induce striped interstitial fibrosis contributing to ESRD. Collagen is the major constituent of extra-cellular matrix protein and the intrarenal expression of the protein is highly elevated in patients with TAC-induced nephrotoxicity (48). Though many types of collagen are expressed in the renal cells, increased deposition of type I collagen is observed in TAC-induced nephrotoxicity (39). In the present study, accumulation of collagen type I in TAC treated cells was found to be reduced in EAF co-treated cells, suggesting the significant role of EAF in alleviating the accumulation of extracellular matrix proteins. 

The major beneficial properties of the plant are attributed to its polyphenol content as well as the flavonoids. Flavonoids are also emerging as dietary supplements to reduce nephrotoxicity and to improve kidney health (49, 50). Hence, EAF was investigated for the active component and quantified by HPLC analysis based on the parameters indicated in the literature. EAF revealed the presence of the bioflavonoid Quercetin (87.5 mg/g), a potent antioxidant and a free radical scavenger. Quercetin is widely distributed in many fruits, vegetables, and plants and is proven to possess many salubrious properties. A previous study has confirmed the presence of Quercetin in *B. diffusa*, which alone and in combination with vitamin E reduced CsA-induced nephrotoxicity, primarily through its antioxidant activity (51). Therefore, in the present study, the nephroprotective activity of EAF of* B. diffusa* may be mainly accredited to the presence of the phytoconstituents Quercetin. 

From the above results, it could be surmised that EAF treatment ameliorates TAC induced nephrotoxicity by lowering the TAC-induced apoptosis & necrosis and ROS production, where the underlying nephroprotective mechanisms of EAF were attributed to its anti-oxidant and anti-apoptotic properties. A previous study has also reported that the ethanolic extract of *B. diffusa* attenuated the lipopolysaccharides (LPS) induced NO production in mouse macrophage (48). Previous studies with the plant extract as well as the current data suggest that co-treatment with the plant decreases the oxidative stress marker induced apoptosis in the cells. Therefore, it can be predicted that the antioxidant potential of EAF plays an important role in reducing TAC-induced mitochondrial injury and apoptosis in the kidney cells. These findings may provide new insight into novel benefits of EAF of BD as a nephroprotective agent. Thus an adjuvant drug therapy, to inhibit apoptosis and trigger the regenerative process through dedifferentiation and proliferation would provide an important strategy to protect the kidney against nephrotoxicity.

**Table 1:** Effect of Ethyl acetate fraction( EAF) co-treatment on tacrolimus (TAC) induced cell cycle arrest and cell death in MDCK cells

**Treatment**	**% of cells in each phase of the cell cycle**			
	**Sub G**_0_ **/G**_1_	**G**_0_ **/G**_1_	**S**	**G**_2_ **/M**
**Control**	5.56%±0.16	75.95±0.05	4.81±0.12	12.50±0.52
**TAC (50 µM)**	27.21±0.75^*^	50.79±0.17^*^	13.25±0.21^*^	8.64±0.54^*^
**TAC + EAF (25 µg/ml)**	12.90±0.31^*^	64.63±0.41^*^	11.10±0.37^*^	10.70±0.14^*^
**TAC + EAF (50 µg/ml)**	8.29±0.07^*^	71.97±0.36^*^	7.31±0.46^*^	12.05±0.31^*^

Data are represented as mean+ SEM of three independent experiments. *P<0.05 for significant difference between treatments in comparison with control

(i) Effect of EAF on the mitochondrial membrane potential in TAC treated cells. Cells were stained with Rhodamine 123 dye and relative fluorescence was measured by flowcytometry. *P-value <0.05 vs TAC-treated group. (ii) Relative optical densities of cytochrome c and β – actin protein were measured in control and treated cells. ***P-value <0.0001. (iii) Immunoblot for cytoplasmic cytochrome c levels. 50 μg of protein was loaded in each lane (a-d). Control (a), TAC (50 µM) (b), TAC (50 µM) + EAF (25 μg/ml) (c), TAC (50 µM) + EAF (50 μg/ml) (d). EAF co-treatment (c&d) treatment decreased the translocation of cytochrome c compared with TAC individually treated cells. (iv) Effect of TAC alone & EAF co-treatment on caspase 3 activity. EAF co-treatment reduced the caspase 3 release significantly. **P-value<0.001. Data are expressed as mean±SEM (n = 3) (A) Control, (B) TAC (50 µM), (C) TAC (50 µM) + EAF (25 μg/ml), (D) TAC (50 µM) + EAF (50 μg/ml)

(i) Effect of tacrolimus (TAC) and Ethyl acetate fraction (EAF) co-treatment on collagen accumulation. The intensity of the red color is directly proportional to the collagen accumulation (a-d). (ii) Quantification of collagen accumulation. Collagen accumulation is significantly reduced in EAF co-treated cells. Data are represented as mean±SEM. *** denotes *P*<0.0001. (A) Control, B) TAC (50 µM), C) TAC (50 µM) and EAF (25 μg/ml), D) TAC (50 µM) and EAF (50 μg/ml)

The chromatographic profiles of (A) Standard at 370 nm and (B) EAF

## Conclusion

The present study demonstrated the dose-dependent protective effect of EAF against TAC induced cytotoxicity in MDCK cell lines. The possible mechanism of the fraction could be the antioxidant potential against TAC induced cytotoxicity, by reducing ROS generation, mitochondrial damage, and the resulting cell death. Thus, the study provides an insight into EAF as an adjuvant therapy against TAC-induced cytotoxicity in kidney cells, hence beneficial for the design of effective therapeutic strategies without compromising the targeted therapy. However the study warrants further *in vivo* studies. 
